# Genetic Modifications to Alter Blood Pressure Level

**DOI:** 10.3390/biomedicines10081855

**Published:** 2022-08-01

**Authors:** Hiroki Ohara, Toru Nabika

**Affiliations:** Department of Functional Pathology, Faculty of Medicine, Shimane University, Izumo 693-8501, Japan; nabika@med.shimane-u.ac.jp

**Keywords:** knock-out, genome-editing, SHR, SHRSP, Dahl SS

## Abstract

Genetic manipulation is one of the indispensable techniques to examine gene functions both in vitro and in vivo. In particular, cardiovascular phenotypes such as blood pressure cannot be evaluated in vitro system, necessitating the creation of transgenic or gene-targeted knock-out and knock-in experimental animals to understand the pathophysiological roles of specific genes on the disease conditions. Although genome-wide association studies (GWAS) in various human populations have identified multiple genetic variations associated with increased risk for hypertension and/or its complications, the causal links remain unresolved. Genome-editing technologies can be applied to many different types of cells and organisms for creation of knock-out/knock-in models. In the post-GWAS era, it may be more worthwhile to validate pathophysiological implications of the risk variants and/or candidate genes by creating genome-edited organisms.

## 1. Introduction

Hypertension is the leading preventable risk factor for cerebro-cardiovascular complications, including heart failure and stroke. Effective anti-hypertensive drugs with different pharmacological actions have been developed; nevertheless, it is deemed that there are 1.28 billion hypertensive patients globally and 0.7 billion or more patients are untreated [1]. Given the resulting mortality and disability as well as the high prevalence, hypertension is still a major public health burden in the world.

It is needless to say that gene-targeted knock-out (KO) and knock-in (KI) or transgenic rodent models have greatly contributed to understanding the pathophysiological basis of hypertension and its vascular complications. In particular, mice have been widely used as the best experimental animal since the gene engineering technique to create KO models was established for over 30 years ago. By contrast, it had been technically difficult to create KO rats for a long time because of the difficulty of rat ES cell culture. Recent advances in genome-editing technologies, however, have made it possible to easily create KO rats similar to mice [2,3]. Given that spontaneous cerebro-cardiovascular disease models, such as Dahl salt-sensitive (SS) and stroke-prone spontaneously hypertensive rats (SHRSP), have been commercially available, a genome-editing strategy using the rat disease models has much potential to clarify the novel pathogenesis of hypertension. In this review, we outlined recent advances in basic research for hypertension using KO and KI or transgenic rodent models to clarify the underlying mechanisms.

## 2. Mouse Models

Essential hypertension is a highly complex pathological condition that is formed by synergistic influences of multiple lifestyles, social, environmental, and genetic factors. Since blood pressure (BP) is collaboratively controlled by various organs and tissues, there are many studies that have investigated tissue (or cell)-specific roles of genes on BP regulation using conventional and conditional KO or transgenic mice. In contrast to rats, no spontaneous hypertensive mouse models have been established; accordingly, angiotensin II (Ang II)-infused models have been widely used to investigate the pathogenesis of Ang II-related hypertension. Deoxycorticosterone acetate (DOCA)-salt or high-salt diet (usually containing 4% or 8% NaCl) models have been also used to investigate the pathogenesis of salt-sensitive hypertension. In this section, we overview proposed mechanisms for controlling BP found in the phenotyping of KO or transgenic mouse models, especially focusing on the findings in the recent decade.

### 2.1. Kidney

The kidney plays pivotal roles in arterial BP regulation by controlling blood volume and plasma electrolyte balance. Activities of the renin–angiotensin–aldosterone system (RAAS) and mineral transporters (Na^+^/H^+^ exchanger; NHE, Na^+^–K^+^–Cl^−^ co-transporter; NKCC, Na^+^–Cl^−^ co-transporter; NCC, epithelial sodium channel; ENaC, etc.) distributed along with nephron are important for physiological BP regulation; thus, genes that may regulate those activities have been widely investigated (Table 1).

Ang II regulates BP via Ang II type 1 receptor (*Agtr1a*, AT1R). As BP lowering effects were observed in proximal tubules (PT) or collecting duct (CD)-specific KO mice [4,5], blockade of AT1R signaling in renal epithelial cells would be a pharmacological target for hypertension therapy. Of note, AT1R-associated protein (*Agtrap*), which is widely distributed along renal tubules, has been found to suppress AT1R signaling by facilitating internalization of AT1R resulting in decreased cell surface expression of AT1R [6,7], suggesting that activation of endogenous AGTRAP has potential to reduce BP. In fact, it has been reported that the renal-specific overexpression and conventional KO mice show lower and higher BP phenotype compared with the wild-type (WT) control, respectively [8,9,10,11]. In contrast to the results in mice, however, the deletion in Dahl SS rats exacerbated renal damage under a 4% NaCl diet condition with no change in BP [12]. Although AGTRAP may play double-edged roles in reno-cardiovascular functions in a context-specific manner, it is a potential candidate gene located in a GWAS loci for BP in humans [12].

Although Ang II is the most well-known bioactive peptide hormone in the RAAS, (pro)renin and Ang-(1-7) produced by angiotensin-converting enzyme 2 (ACE2) are also known to regulate BP via its specific receptors. The (Pro)renin receptor (PRR) that specifically recognizes both prorenin and renin was cloned by Nguyen et al. in 2002 [13]. In the kidney, PRR is mainly expressed in renal vasculature, PT and distal tubules (DT), and CD and enhances the catalytic activity of (pro)renin that converts Ang I to Ang II, resulting in an increase in Ang II production [14]. Consistent with the physiological function of PRR, decreases in BP elevation induced by Ang II infusion have been observed in both tubular- and CD-specific KO mice through inhibition of ENaC activation [15,16,17]. Ang-(1-7) generated by mainly ACE2 is a vasoactive peptide that induces a vasodilation response by binding to Mas receptor [18]. Therefore, ACE2-Ang-(1-7)-Mas axis exerts a counteracting effect on Ang II that causes BP elevation. Ni et al. reported that conventional double KO of both ACE2 and Mas receptor in mice caused greater Ang II-induced BP elevation when compared with the WT littermates [19]. In addition, they also showed that the dual deletion of ACE2 and Mas receptor worsened hypertensive nephropathy, suggesting that ACE2-Ang-(1-7)-Mas receptor axis has protective roles in both the development of hypertension and the resulting hypertensive kidney injury.

Tubuloglomerular feedback (TGF) is an important physiological system to regulate long-term BP by sensing blood volume and electrolyte balance at the level of juxtaglomerular apparatus in each nephron [20]. Accumulating evidence has shown that local activities of renal oxide synthases (NOS), which produce a major chemical vasodilator NO, play an important role in the regulation of the TGF system. NOS families are composed of three isoforms, i.e., neuronal NOS (nNOS, encoded by *Nos1*), inducible NOS (iNOS, *Nos2*), and endothelial NOS (eNOS, *Nos3*). Although all the three isoforms are expressed in the kidney, *Nos1* and *Nos3* are thought to be major isoforms that physiologically participate in the TGF because of low baseline expression of *Nos2*. Interestingly, Lu et al. showed that macula densa-specific deletion of *Nos1* exacerbated a high-salt diet-induced BP elevation under a condition of Ang II infusion accompanied by reduced glomerular filtration rate (GFR) and Na^+^ excretion [21]. It was also reported that local NOS1 activity at the macula densa contributed to a sex difference in BP response to Ang II [22]. Moreover, Hyndman et al. and Gao et al. have investigated renal-specific roles of NOS1 and NOS3 on BP regulation using CD-specific and nephron-specific KO mice, respectively [23,24]. They suggested that deletion of the two isoforms caused greater high-salt-induced BP elevation by enhancing ENaC [25] and NCC activities in the tubular cells, respectively.

Pathophysiological roles of NEDD4-2 (encoded by *Nedd4l*) and with-no-lysine kinases 1 and 4 (*Wnk1* and *Wnk4*) in (salt-sensitive) hypertension have been well-investigated in humans as well as in rodent models. NEDD4-2 is an E3 ubiquitin ligase that ubiquitylates ENaC to down-regulate its cell surface expression and activity [26]. Although NEDD4-2 was initially found as a ENaC-specific regulator in the kidney [25], Ronzaud et al. reported that NEED4-2 also regulated NCC activity and its renal tubule-specific deletion caused salt-dependent hypertension [27]. Consequently, NEED4-2 is involved in the pathogenesis of salt-sensitive hypertension through the two-independent pathways that controls renal Na^+^ homeostasis. WNK1 and WNK4 are known to be responsible genes of pseudohypoaldosteronism type 2 (PHA2) that is caused by large deletions in intron 1 of WNK1 or gain-of function mutations in WNK4 [28]. Mechanistically, WNKs phosphorylate SPAK/OSR1, thereby activating NCC in the DT and resulting in increased Na^+^ reabsorption and salt-sensitive hypertension [28,29]; however, the molecular network may be a little complicated as a paradoxical role of kidney-specific WNK1 lacking a kinase domain on the development of salt-sensitive hypertension was reported [30]. Moreover, Mu et al. suggested a unique pathway involving salt-sensitive hypertension caused by epigenetic down-regulation of WNK4 [31]. In this context, kelch-like protein 3 (KLHL3) and cullin 3 (CUL3), which are the E3 ubiquitin ligase complex to degrade WNK, have also received much attention as target molecules to prevent salt-sensitive hypertension [28,29].

Unlike the local mechanisms in the kidney described above, Pan et al. uniquely identified the liver–kidney and liver–adipocytes axis to control BP via a hepatocytes-producing hormone, fibroblast growth factor 21 (FGF21), which has pleiotropic effects on glucose and lipid metabolism [32]. They found that FGF21 augmented peroxisome proliferator-activated receptor γ (PPARγ)-mediated activation of ACE2 in both the kidney and adipocytes; thereby, an increase in Ang-(1-7) production reduced both BP and vascular injury. Because FGF21 production was stimulated by Ang II, the FGF21–ACE2 axis may counteract Ang II-induced hypertension and the vascular injury. This might be a key mechanism in obesity-related hypertension.

Besides the above, multiple mechanisms have been proposed such as by circadian clock- [33,34], osmotic stress- [35], and genome-wide association study (GWAS)-related genes [36,37] as well.

**Table 1:** Target molecules in kidney.

Targets	Type of Genetic Modification	Models	Phenotypes	References
AGTRAP (angiotensin II receptor-associated protein, *Agtrap*)	Renal tubule-specific overexpression	Ang II	↓BP, ↓NCC and αENaC activities	Wakui et al. [8]
	Conventional KO	Ang II	↑BP, ↑ENaC activity	Ohsawa et al. [9]
	Conventional KO	5/6 nephrectomy	↑BP, ↑plasma volume, ↑αENaC and TNF-α expression	Kobayashi et al. [10]
	Proximal tubule-specific KO	Ang II	No differences in basal BP, pressor response to Ang II, and cardiac hypertrophy	Kinguchi et al. [11]
PRR ((Pro)renin receptor, *Atp6ap2*)	Tubular-specific KO	Ang II	↓BP, ↓Na^+^ retention, ↓αENaC expression	Ramkumar et al. [15]
	Collecting duct-specific KO	Ang II	↓BP, ↓urinary renin and αENaC activities	Peng et al. [16]
	Collecting duct-specific KO	Ang II	↓BP (basal and Ang II), ↓α/γENaC activation, ↓urinary Ang II and renin levels	Prieto et al. [17]
ACE2 (angiotensin-converting enzyme-2, *Ace2*), Mas receptor (*Mas1*)	Conventional double KO	Ang II	↑BP, ↑ renal injury, ↑serum Cr, ↓Cr clearance	Ni et al. [19]
NOS1 (NO synthase 1, *Nos1*)	Macula densa-specific KO	Ang II + high-salt diet	↑BP, ↑ tubuloglomerular feedback response, ↓GFR, urine flow, and N^+^ excretion	Lu et al. [21]
	Macula densa-specific KO	Ang II	Diminished sex difference in Ang II-induced BP, tubuloglomerular feedback response, and natriuretic response	Zhang et al. [22]
	Collecting duct-specific KO	High-salt diet	↑BP, ↓urine output, ↓Na^+^, Cl^−^, and NOx excretion	Hyndman et al. [23]
NOS3 (NO synthase 3, *Nos3*)	Doxycycline-inducible nephron-specific KO	High-salt diet	↑BP, ↑Na^+^ retention, ↑NCC activation	Gao et al. [24]
NEDD4-2 (*Nedd4l*)	Tetracycline-inducible tubule-specific KO	High-salt diet	↑BP, ↑β/γENaC and ROMK expression, ↑NCC activation, hypercalciuria	Ronzaud et al. [27]
WNK1 (with-no-lysine kinase 1, *Wnk1*)	Kidney-specific overexpression of the kidney-specific isoform	No treatment	↓BP, ↑plasma Ang II and aldosterone, ↓NCC and NKCC2 activation	Liu et al. [30]
	Kidney-specific KO (targeted deletion of the first exon of the kidney-specific isoform)	High-salt diet	↑BP, ↑Na^+^ retention, ↑NCC and NKCC2 activation	Liu et al. [30]
FGF21 (fibroblast growth factor 21, *Fgf21*)	Conventional KO	Ang II	↑BP, ↑vascular hypertrophy and fibrosis, ↓vascular relaxation, ↓plasma/adipose ACE2 and Ang-(1-7), ↑plasma/adipose Ang II	Pan et al. [32]
BMAL1 (brain and muscle ARNT-like 1, *Arntl*)	Kidney-specific KO	No treatment (or K^+^-restricted diet)	↑BP, ↓Na^+^ retention under K^+^-restricted diet	Crislip et al. [33]
Per1 (period 1, *Per1*)	Distal nephron-specific KO	DOCP-salt	↑BP, ↑Na^+^ retention, ↑plasma aldosterone, ↑medullary endothelin-1	Douma et al. [34]
NFAT5 (nuclear factor of activated T-cells 5, *Nfat5*)	Doxycycline-inducible tubular cell-specific KO	High-salt diet	↑BP, hypernatremia, polyuria, ↓Na^+^ excretion, ↑ENaC expression	Hiramatsu et al. [35]
HSD11β2 (11β-hydroxysteroid dehydrogenase, *Hsd11b2*)	Kidney-specific KO	No treatment	↑BP, ↑αENaC and NCC activation	Ueda et al. [36]
NPR-C (natriuretic peptide receptor-C, *Npr3*)	Conventional KO	Ang II	↓BP, ↑diuretic and natriuretic response, ↓NCC activation via WNK4/SPAK	Shao et al. [37]
	Tubule-specific KO	Ang II	↓BP, ↓NCC activation via WNK4/SPAK	Shao et al. [37]

### 2.2. Vasculatures

Table 2 summarizes target genes in vasculatures and the representative phenotypes described below. Peripheral vascular tone is one of the primary factors to control BP. Two primary cell types, i.e., endothelial cells (ECs) and vascular smooth muscle cells (VSMCs), play major roles in the regulation of the vascular tone mainly through production of vasodilators (NO, etc.) or vasoconstrictors (endothelin-1, etc.) and sympathetic vasoconstriction, respectively. Endothelial NOS (eNOS, NOS3) predominantly generates NO from L-arginine in ECs; thereby, the released NO activates NO-sensitive guanylyl cyclase (NO-GC) in VSMCs to increase cytosolic cGMP, then the activated cGMP-dependent protein kinase (PKG) induces smooth muscle relaxation. The NO-GC/cGMP/PKG signaling is indispensable for NO-dependent BP regulation as spontaneous BP elevation was found in VSMC-specific NO-GC deficient mice [38].

In addition, NO-independent pathways that stimulate cGMP/PKG also exist. Natriuretic peptides, which are composed of A- (atrial; ANP), B- (brain; BNP), and C-type (CNP), are well-studied vasoactive peptides that exert vasodilation via direct activation of the transmembrane receptor NPR1 (natriuretic peptide receptor 1, also known as guanylyl cyclase-A; GC-A) or NPR2 (also known as GC-B). Among the three members, CNP is secreted from ECs and specifically bind to NPR2, whereas ANP and BNP are cardiac peptides targeting NPR1 [39]. In addition, CNP is thought to be an autocrine/paracrine factor in the circulation system because of the relatively low plasma concentration compared with ANP and BNP [40]. Several recent reports have uncovered the detailed mechanisms of NPR1/2-mediated vasodilation.

Nakao et al. showed that EC-specific CNP KO mice had higher BP compared with WT control independently of NO production, whereas VSMC-specific NPR2 KO had unaltered BP [41]. On the other hand, Špiranec et al. thereafter reported that the deletion in ‘precapillary arteriole SMCs and capillary pericytes’ caused BP elevation in mice accompanied by an impaired CNP-induced vasodilatory response [42]. Collectively, these results indicate that EC-derived CNP acts on precapillary arteriole SMCs and capillary pericytes as well as ECs to lower peripheral vascular resistance and BP through an NO-independent manner. It is of note that a CNP-induced vasodilatory response in mesenteric arteries was also impaired in VSMC-specific KO mice by Nakao et al.; nevertheless, BP of the KO mice was compatible with that of WT [41]. Conflicting results for the BP phenotype between the two VSMC-specific KO models may be partly due to the difference in promoters driving Cre expression (*sm22* [41] or *Pdgf-rb* [42] promoter) to create the conditional NRP2 KO mice. Furthermore, it was very recently reported that EC-specific, but not VSMC-specific, deletion of NPR1 diminished BP reduction by intravenous ANP administration [43].

Intracellular Ca^2+^ mobilization is a key modulator to induce both NOS-mediated vascular relaxation by EC and VSMC contraction; thus, cell-type specific genetic modification is necessary to clarify functional implications of the target molecules on vascular responses. Stromal interaction molecule 1 (*Stim1*) is an endoplasmic reticulum (ER) resident transmembrane protein that senses Ca^2+^ store in ER lumen via its N-terminal EF hand motif. When the Ca^2+^ store is depleted, STIM1 moves toward the cytoplasmic membrane and opens the target Ca^2+^ channels, ORAI1, and transient receptor potential families (TRPs) to elicit Ca^2+^ entry into the cytosol (store-operated Ca^2+^ entry; SOCE) [44]. STIM1 is expressed in broad cell types including ECs and VSMCs and plays key roles in the maintenance of intracellular Ca^2+^ homeostasis [43]. Kassan et al. revealed that VSMC-specific deletion of *Stim1* ameliorated Ang II-induced hypertension with decreased vascular ER stress [45]. By contrast, a significant increase in nighttime BP was observed in EC-specific *Stim1* KO mice that showed decreased NO production and an EC-dependent vasodilation phenotype in vitro [46]. These studies suggest that STIM1 exerts an opposite role in the regulation of vascular tone in the two different cells. Interestingly, we found that the stroke-prone spontaneously hypertensive rat (SHRSP) had a premature stop codon in this gene that caused the expression of truncated STIM1 with decreased SOCE activity [47,48]. Although the recovery of STIM1 function in SHRSP by CRISPR-Cas9-mediated gene KI did not alter the BP [49], systemic impairment of SOCE activity would have important implications for the pathogenesis of hypertensive end-organ damage in SHRSP independently of the BP phenotype. Phenotyping of the KI rat model is currently in progress; the findings will be described elsewhere.

Fluid shear stress is an important mechanical stimulus that physiologically enhances NO production by ECs to maintain vascular integrity. Increasing evidence has shown that a mechanosensitive cation channel PIEZO1 on ECs mediates laminal flow-dependent activation of purinergic P2Y2 receptor, thereby activating PI3K/Akt signaling to phosphorylate NOS3 [50]. Recently, an alternative pathway mediated by the PIEZO1adrenomedullin (ADM) axis was reported [51]. ADM is a circulatory vasodilator and diuretic and natriuretic peptide that is mainly produced by ECs [52]. Iring et al. showed that PIEZO1 enhanced endothelial ADM secretion, then the secreted ADM bound to calcitonin receptor-like receptor (CALCRL) on ECs by an autocrine/paracrine fashion. ADM-CALCRL complex activates its adjacent adenylyl cyclase; thereby, cAMP-dependent protein kinase (PKA) phosphorylates and activates NOS3, resulting in NO-dependent vasorelaxation [50]. Actually, it is of interest that all EC-specific single KO of ADM, CALCRL, and Gαs, which is the downstream G protein of CALCRL, in mice caused apparent BP elevation. This finding indicates an essential role of PIEZO1-ADM signaling on controlling vascular tone and BP at a resting condition.

Prostaglandins (PGs) are endogenous lipid mediators that have multiple bioactivities such as uterine contraction, platelet aggregation, and bronchodilation and is generated from arachidonic acid by catalytic activities of cyclooxygenases (COXs). PGs are also involved in BP regulation as nonsteroidal anti-inflammatory drugs (NSAIDs), which block COX activity, have hypertensive side effects [53]. Among the known PGs, PGE_2_ is a major prostanoid that affects BP both positively and negatively via its specific receptor EP1-4 [54,55]. Thus far, it was shown that EP1 and EP3 mediate vasoconstrictive response, while EP2 and EP4 lead to vasodilation [53]. The diverse effects of PGE_2_ on vascular functions may be due to the characteristic of tissue distribution of the receptors. Recently, Xu et al. reported that EC-specific KO and overexpression of EP4 resulted in higher and lower BP compared with control mice, respectively [56], under both basal and high-salt diet conditions. Physiological roles of PGs on BP regulation may be still controversial; however, the recent report clearly indicates a hypotensive potential of PGE_2_-EP4 signaling via enhancing NO production in ECs.

**Table 2:** Target molecules in vasculatures.

Targets (*Official Symbols*)	Type of Genetic Modification	Models	Phenotypes	References
NO-GC (NO-sensitive guanylyl cyclase, *Gucy1b1*)	Tamoxifen-inducible VSMC-specific KO	No treatment	↑BP, ↓NO-induced vasorelaxation	Groneberg et al. [38]
CNP (C-type natriuretic peptide, *Nppc*)	EC-specific KO	No treatment	↑BP, ↓acetylcholine- and endothelium-dependent relaxation, ↑Endothelin-1 and Ace expression in ECs	Nakao et al. [41]
NPR2 (natriuretic peptide receptor 2, *Npr2*)/Guanylyl cyclase-B (GC-B)	VSMC-specific KO	No treatment	No difference in BP, ↓CNP-induced relaxation in mesenteric arteries	Nakao et al. [41]
	Tamoxifen-inducible EC-specific KO	No treatment	↑BP, ↓cGMP production	Špiranec et al. [41]
NPR1 (natriuretic peptide receptor 1, *Npr1*)	EC-specific KO	No treatment	Loss of EC-dependent BP reduction by ANP, unaltered NO production, K^+^ channel-mediated hyperpolarization in EC	Tokudome et al. [43]
STIM1 (stromal interaction molecule 1, *Stim1*)	VSMC-specific KO	Ang II	↓BP, ↓cardiac hypertrophy, ↓perivascular fibrosis, ↓endothelial dysfunction	Kassan et al. [45]
	EC-specific KO	No treatment	↑BP (nighttime), ↓NO production, ↓endothelium-dependent relaxation	Nishimoto et al. [46]
ADM (adrenomedullin, *Adm*), CALCRL (calcitonin receptor-like, *Calcrl*), G_αs_ (GNAS (guanine nucleotide binding protein, alpha stimulating) complex locus, *Gnas*)	Tamoxifen-inducible EC-specific KO	No treatment	↑BP, ↓eNOS activation, ↓flow-induced vasorelaxation	Iring et al. [51]
EP4 (prostaglandin E2 receptor, *Ptger4*)	EC-specific KO	No treatment, high-salt diet, Ang II	↑BP, ↓NO production, ↓vasorelaxation response	Xu et al. [56]
	EC-specific overexpression	No treatment, high-salt diet	↓BP, ↑eNOS activation, ↑NO production	Xu et al. [56]

### 2.3. Immunity

A growing body of evidence has emerged in the last decade suggesting the pathogenic aspects of innate and adaptive immune responses on the development and progression of hypertension and hypertensive end-organ damages. The possible mechanisms have been well reviewed [57,58,59]; herein, we shortly highlight recent findings on this topic (Table 3). Among various subpopulations of immune cells, previous reports indicate that T cells especially have diverse contributions to the etiology [56]. In particular, CD4^+^- and regulatory T cell (Treg)-mediated pathological cascades have been raised in several recent studies.

Hydrogen sulfide (H_2_S) is a cardioprotective endogenous gaseous mediator that is generated by three major enzymes: cystathionine beta synthase (CBS), cystathionine gamma lyase (CSE), or 3-mercaptopyruvate sulfurtransferase [60]. Although it was reported that a conventional CSE KO mice showed an age-dependent increase in BP [61], Cui et al. revealed that CD4^+^ T cell-specific deletion of CSE was sufficient to induce greater BP in mice under both physiological and Ang II-treated conditions [62]. Mechanistically, they suggest that CSE-derived H_2_S activates liver kinase B1 (LKB1)-PKA signaling and the resulting activation of Treg attenuates vascular and renal inflammation, thereby preventing BP elevation. In addition, Sun et al. reported that mineralocorticoid receptor (MR) deficiency in CD4^+^ T cells ameliorated Ang II-induced BP elevation and vascular and renal damage in mice [63]. In contrast to the KO model, the MR overexpression exacerbated the increase in BP after Ang II infusion; however, IFN-γ-neutralizing antibodies could abolish the deleterious effect, suggesting that IFN-γ produced by infiltrated T cells was a key cytokine link between MR signaling in CD4^+^ T cells and the resulting hypertension.

For Treg, a detailed pathological mechanism caused by a microRNA function has been proposed. MicroRNAs (miRs) are involved in numerous (patho)physiological conditions by controlling gene expression mainly at a translational level, and among the identified miRs, miR-31 has multifaceted roles in regulation of immune responses [64]. Interestingly, Li et al. reported that Ang II-induced BP elevation and vascular and renal damage were reduced in mice lacking miR-31 in Treg compared with control mice, which were accompanied by increased Treg differentiation [65]. Furthermore, the opposite phenotypes were observed in mice with Treg-specific deletion of protein phosphatase 6c (*Ppp6c*), a direct target of miR-31, suggesting that *Ppp6c* had potential to improve Ang II-induced hypertension [64]. This may be a novel posttranslational mechanism that worsens hypertensive phenotypes through an overexpression of a specific microRNA that regulates Treg functions.

AT1R, an Ang II receptor, is widely expressed in immune cells [66]. It has been shown that deletion of AT1R on T lymphocytes or macrophages do not affect BP even under an Ang II-infused condition [67,68]. In contrast to the previous findings, Lu et al. revealed that the deletion on CD11c^+^ myeloid cells (dendritic cells; DCs) in mice with chronic Ang II infusion resulted in increased BP, renal infiltration of inflammatory cells (memory T, CD40^+^ DCs), and Na^+^ retention with greater β/γENaC expression [69]. It is of interest that AT1R on DCs exerts a cardioprotective role in spite of harmful effects of Ang II on renal and cardiovascular functions. Moreover, Sag et al. showed that mice with myeloid cell-, but not endothelial cell-, specific deletion of NADPH oxidase 2 (NOX2), which is a superoxide-generating enzyme, had lower basal BP compared with control mice [70].

Beside the above, the pathophysiological actions of C-C motif chemokine receptor 7 (CCR7) [71], toll-like receptor 3/4 (TLR3/4) [72], placental growth factor (PlGF) [73], complement C3a/C5a receptors (C3aR/C5aR) [74], T cell receptor delta chain (TCRδ) [75], and interleukin-1 receptor type 1 (IL-1R1) [76] have been also proposed using conventional KO mouse models. Overall, accumulated evidence has commonly suggested pathophysiological connections between immune responses and renal dysfunction on the development of hypertensive conditions. Clinical perspectives of anti-inflammatory therapies targeting specific cytokines were also discussed [77].

**Table 3:** Target molecules in immune system.

Targets (*Official Symbols*)	Type of Genetic Modification	Models	Phenotypes	References
CSE (Cystathionine γ lyase, *Cth*)	CD4^+^ T cell-specific KO	Ang II	↑BP, ↑blood and renal Treg, ↑renal and peripheral adipose tissue CD4^+^/CD8^+^ T	Cui et al. [62]
MR (mineralocorticoid receptor)/nuclear receptor subfamily 3, group C, member 2 (*Nr3c2*)	CD4^+^ T cell-specific KO	Ang II	↓BP, ↓renal/vascular damage, ↓IFNγ-producing T cell	Sun et al. [63]
	CD4^+^ T cell-specific overexpression	Ang II	↑BP	Sun et al. [63]
MicroRNA-31 (miR-31, *Mir31*)	Conventional and Treg-specific KO	Ang II	↓BP, ↑Treg differentiation,↓renal and vascular injury	Li et al. [65]
Ppp6c (protein phosphatase 6c, *Ppp6c*)	Treg-specific KO	Ang II	↑BP, ↓Treg differentiation,↑renal injury	Li et al. [65]
AT1R (angiotensin II receptor type 1, *Agtr1a*)	CD11c^+^ cell-specific KO	Ang II	↑BP, ↑renal memory T and CD40^+^ DC, ↑ENaC	Lu et al. [69]
NOX2 (NADPH oxidase 2, *Cybb*)	Myeloid cells-specific KO	No treatment	↓BP, ↑NO bioavailability	Sag et al. [70]
		Ang II	No effect on BP	Sag et al. [70]
CCR7 (C-C motif chemokine receptor 7, *Ccr7*)	Conventional KO	Ang II	↓BP, ↑renal CD8^+^ T, ↓renal draining lymph node CD4^+^ T and CD8^+^ T	Wen et al. [71]
TLR3/4 (toll-like receptor 3/4, *Tlr3/4*)	Conventional KO	Ang II	↓BP and cardiac hypertrophy in TLR3 KO, ↓cardiac hypertrophy in TLR4 KO	Singh et al. [72]
PIGF (placental growth factor, *Pgf*)	Conventional KO	DOCA-salt	↓BP, ↓renal damage and T cell infiltration	Perrotta et al. [73]
C3aR/C5aR (complement 3a and 5a receptors, C3ar1/C5ar1)	Conventional double KO	Ang II	↓BP, ↑renal Treg, ↓renal/vascular remodeling	Chen et al. [74]
TCRδ (T cell receptor delta chain, *Tcrd*)	Conventional KO	Ang II	↓BP, ↓endothelial dysfunction	Caillon et al. [75]
IL-1R1 (IL-1 receptor type 1, *Il1r1*)	Conventional KO	Ang II	↓BP, ↓NKCC2 activity	Zhang et al. [76]

### 2.4. Other Organs and Tissues (Brain, Adipocyte, and Adrenal Gland)

Table 4 summarizes target genes in other major organs and tissues and the representative phenotypes. Pathological implications of brain RAAS on hypertension have been well investigated [78]. Based on the distribution of RAAS components in the brain, it has been verified that brain RAAS activity can induce BP elevation independently of renal RAAS function by using cell type-specific transgenic mice targeting AGT and/or renin [79,80,81]. Consistent results were observed in DOCA-salt mice with neuron-specific deletion of PRR that exhibited decreases in BP and brain Ang II production [82].

Pathological relationships between salt intake and hypertension have been long suggested in humas as well as in rodents; however, the precise mechanism remains elusive. In this context, it is noteworthy that Nomura et al. reported that the Na_x_ channel expressed in specific glial cells in the organum vasculosum lamina terminalis (OVLT) functioned as the brain sensor detecting [Na^+^] increase in the body and that deletion of Na_x_ diminished salt-induced hypertensive phenotype [83]. Concerning this, neuronal 11β-hydroxysteroid dehydrogenase type2 (*Hsd11b2*), which encodes a corticosterone-producing enzyme, and PRR have been proposed to be involved in both the development of salt-sensitive hypertension and sodium appetite [84,85]. In addition, PRR deficiency in adipocytes was pathologically implicated in a high-fat diet-induced BP increase in male mice but not in female mice [86].

The adrenal gland is a major endocrine organ that plays a pivotal role in BP regulation and fluid and electrolyte homeostasis via production of steroid hormones and catecholamines. The two-pore domain K^+^ channels (TASKs) expressed in zona glomerulosa (zG) cells down-regulate the production of aldosterone in the cells [87]. Guagliardo et al. showed that zG cell-specific deletion of TASK-1 and -3 caused autonomous hyperaldosteronism and chronic BP elevation in mice [88]. In addition, Mathar et al. reported that mice lacking transient receptor potential melastatin 4 (TRPM4) had chronically increased BP with exaggerated sympathetic tone [89]. TRP families are non-selective cation channels that are involved in many physiological processes and are regarded as potential targets for drug design for various diseases [50]. According to the report by Mathar et al., TRPM4 deficiency increases catecholamine release from chromaffin cells and thereby augments sympathetic tone resulting in a continuous BP elevation.

It is generally known that plasma concentrations of adrenal gland-derived steroid hormones are controlled by the physiological circadian rhythm of adrenocorticotropic hormone (ACTH) secretion. Circadian clock genes, cryptochrome-1 and -2 (Cry-1 and -2), play key roles in this mechanism by direct regulation of *Hsd3b6* expression encoding an aldosterone-producing enzyme, 3β-hydroxysteroid dehydrogenase-isomerase (3β-HSD). Therefore, Cry-1 and Cry-2 KO mice exhibited salt-sensitive hypertension under a high-salt diet condition due to constitutive activation of 3β-HSD such asthe DOCA-salt model [90].

Multiple genetic and physiological mechanisms as thus far described are complicatedly involved in the pathogenesis of hypertension. Furthermore, the pathogenic roles of epigenetic modifications [91,92,93], microbiota/metabolome [94,95,96], and sympathetic overactivity [97,98,99] in cardiovascular disease have been also discussed.

**Table 4:** Target molecules in brain, adipocyte, and adrenal gland.

Organs, Tissues	Targets (*Official Symbols*)	Type of Genetic Modification	Models	Phenotypes	References
Brain	Human AGT (angiotensinogen, *AGT*)	Glial-specific overexpression	No treatment	↑BP,↑salt preference	Morimoto et al. [79]
	Human REN (renin, *REN*)	Glial- and neuron-specific overexpression	No treatment	↑BP, ↑salt preference	Morimoto et al. [80]
	Human AGT (angiotensinogen, *AGT*)	Glial-specific KO	No treatment	↓BP	Sherrod et al. [81]
	PRR ((Pro)renin receptor, *Atp6ap2*)	Neuron-specific KO	DOCA-salt	↓BP, ↓brain Ang II production, ↓cardiac and vasomotor sympathetic tone	Li et al. [82]
	Na_x_ (sodium channel, voltage-gated, type VII, alpha, *Scn7a*)	Conventional KO	High-salt diet	↑BP	Nomura et al. [83]
	HSD11β2 (11β-hydroxysteroid dehydrogenase, *Hsd11b2*)	Neuron-specific KO	High-salt water	↑BP, ↑salt preference	Evans et al. [84]
Adipocytes	PRR ((Pro)renin receptor, *Atp6ap2)*	Adipocyte-specific KO	High-fat diet	↑BP (basal and high fat diet-induced), ↑glucose tolerance, ↓diet-induced obesity	Wu et al. [86]
Adrenal gland	TASK-1/3 (potassium channel, subfamily K, member 3/9, *Kcnk3/9*)	Zona glomerulosa cells-specific KO	No treatment	↑BP	Guagliardo et al. [88]
	TRPM4 (transient receptor potential cation channel, subfamily M, member 4, *Trpm4*)	Conventional KO	No treatment	↑BP, ↑plasma epinephrine, ↑urinary catecholamine metabolites	Mathar et al. [89]
	Cry1/2 (cryptochrome-1/2, *Cry1/2*)	Conventional KO	High-salt diet	↑BP, ↑increased expression and activity of 3β-HSD	Doi et al. [90]

## 3. Rat Models

Rats are the generally used experimental animal the same as mice and have some advantages compared with mice such as large body and tissue size and physiological properties similar to those in humans. Despite the advantages, mice have been more frequently used than rats; this is probably due to, except for higher experimental costs than mice, the technical difficulty of creating KO/KI rats. However, genome-editing technologies, i.e., zinc finger nuclease (ZFN), transcription activator-like effector nuclease (TALEN), and clustered regularly interspaced short palindromic repeats (CRISPRs)-associated proteins 9 (CRISPR-Cas9), made it possible to also create KO/KI rats easily. In 2009, Geurts et al. first reported the creation of KO rats by ZFN [100]. Thereafter, a growing body of literature has emerged in the last decade reporting phenotypes of KO/KI rats including genetic hypertensive models as below [101,102].

### 3.1. KO Models of SHR and SHRSP

SHR (spontaneously hypertensive rat) is a representative genetically hypertensive model that was established by selective breeding of rats with relatively high blood pressure in an outbred colony of Wistar rats that had been maintained in Kyoto University. SHRSP (stroke-prone SHR) is a substrain of SHR that genetically develops more severe hypertension and stroke. Despite the fact that both strains have been widely used for clarifying the responsible genes and the underlying mechanisms of hypertension and its complications [103,104,105], the literature evaluating cardiovascular phenotypes by using KO/KI models are still scant (Table 5).

SHR is a useful model for hypertensive cardiac hypertrophy [106]. A quantitative trait locus (QTL) related to the left ventricular hypertrophy was previously mapped on chromosome (Chr) 8 by phenotyping of congenic strains between SHR and normotensive Brown Norway (BN) rats [107]. Liška et al. identified promyelocytic leukemia zinc finger (*Plzf*) as a candidate gene on the cardiac QTL and showed that the deletion in SHR did not alter the BP but ameliorated cardiac hypertrophy and fibrosis [108].

Complement 3 (C3) that is overexpressed in aortic smooth muscle cells of SHR has been proposed as a candidate gene responsible for the development of hypertension in this model [109]. Mechanistically, C3-C3a receptor signaling accelerates a change in the characteristic of VSMC and glomerular mesangial cells from contractile to synthetic phenotype via activation of Krüppel-like factor 5 (KLF5) that is a transcription factor to induce the synthetic phenotype of mesenchymal cells [110,111]. Negishi et al. revealed that the C3 deficiency mitigated a salt-sensitive BP elevation and renal injury with decreased renal Ang II level and urinary catecholamine excretion [112].

Rubattu et al. previously identified a QTL on Chr 1 responsible for the susceptibility to salt-induced stroke by a linkage analysis F2 cross between SHR and SHRSP [113]. They identified NADH dehydrogenase (ubiquinone) 1 subunit C2 (*Ndufc2*), encoding a component of the electron transport chain, as a plausible candidate gene in the stroke QTL, then proved that the heterozygous deletion in SHR by ZFN strongly exacerbated the stroke susceptibility with increased oxidative stress and inflammation both in vitro and in vivo [114].

Besides the above, we recently created peroxiredoxin 2 (*Prdx2*) KO SHR to investigate whether the deletion of an antioxidant gene exacerbates cerebro-cardiovascular phenotypes of SHR [115]. Consequently, *Prdx2* KO SHR had greater basal BP compared with WT SHR. Furthermore, the lifespan of *Prdx2* KO SHR under a salt loading condition was shorter than that of WT SHR despite no difference in BP after salt loading between the KO and the WT. No apparent inter-strain differences were found in histopathological evaluation for brain, heart, and kidney lesions, and therefore, the reason for the short life span of *Prdx2* KO SHR under the salt loading condition remains fully unknown.

Lectin-like oxidized low-density lipoprotein receptor-1 (LOX-1) is an endothelial scavenger receptor that is closely involved in the pathogenesis of atherosclerosis [116]. Recently, Liang et al. reported that LOX-1 deficiency had a protective role in spontaneous brain damage in SHRSP with no significant change of BP [117].

We previously found a QTL on Chr1 that affected exaggerated sympathetic responses to the stress of SHRSP by genetic analysis of congenic lines between SHRSP and normotensive Wistar-Kyoto rat (WKY) [118]. Among the genes in the QTL region, stromal interaction molecule 1 (*Stim1*) with a nonsense mutation in SHRSP was identified as a promising candidate ([47], see also Section 2.2 Vasculatures). As STIM1 plays a key role in Ca^2+^ homeostasis in the body, we expected that the *Stim1* mutation was a genetic determinant responsible for cerebro-cardiovascular traits; however, no significant differences were observed in the sympathetic stress responses as well as age-dependent changes in BP between *Stim1* KI SHRSP and SHRSP, i.e., with WT and mutant allele for *Stim1*, respectively [49]. Phenotyping of the *Stim1* KI SHRSP is currently in progress, and the results will be described elsewhere.

### 3.2. KO Models of Dahl SS

Dahl salt-sensitive (SS) rats originate from a closed colony of Sprague-Dawley (SD) rats and are widely used as a salt-sensitive hypertension model that develop severe hypertension (>200 mmHg) and the complications such as hypertensive kidney injury and heart failure when fed high-salt diets [119]. SS/Jr and DSS/N strains have been separately established by Rapp and Iwai, respectively. Compared with SHR and SHRSP, multiple KO/KI models with SS/Jr genetic backgrounds have been actively created (Table 5).

In 2011, Moreno et al. first reported the phenotype of renin KO SS/Jr, in which a severe decrease in basal BP and abnormal kidney morphologies were observed [120]. Thereafter, a growing literature has shown pathophysiological implications of multiple genes on cardiorenal disease traits in SS/Jr [121,122,123,124,125,126,127,128,129,130,131,132,133]. Among them, pleckstrin homology domain containing family A member 7 (*Plekha7*) is a plausible candidate gene for essential hypertension identified by GWAS. A risk variation on *Plekha7*, encoding an adherence junction protein [134], for elevated systolic BP has been found in multiple human populations [135,136,137,138,139]. In this context, Endres et al. created SS/Jr lacking the functional domain of *Plekha7* by ZFN and revealed that the *Plekha7* functional KO SS/Jr had significantly lower BP and renal and cardiac damage under the 8% high-salt diet condition [123]. This is a meaningful study that verified a direct effect of the GWAS gene on the hypertensive phenotype in a genetic rat model with salt sensitivity.

**Table 5:** Target molecules in rat models.

Strains	Targets (*Official Symbols*)	Methods	Phenotypes	References
SHR/OlaIpcv	Plzf (promyelocytic leukemia zinc finger, *Zbtb16*)	TALEN	↑cardiomyocyte hypertrophy and fibrosis	Liška et al. [108]
SHR/NCrl	Ndufc2 (NADH dehydrogenase (ubiquinone) 1 subunit C2, *Ndufc2*)	ZFN	No effect on BP, ↑salt-induced stroke susceptibility, oxidative stress, and inflammatory signaling	Rubattu et al. [113]
SHR/Izm	C3 (complement 3, *C3*)	ZFN	↓salt-induced BP, ↓renal Ang II level, ↓urinary catecholamine excretion	Negishi et al. [112]
	Prdx2 (peroxiredoxin 2, *Prdx2*)	CRISPR-Cas9	↓basal BP, ↑life span under salt loading condition	Mahal et al. [115]
SHRSP/Izm	LOX-1 (lectin-like oxidized low-density lipoprotein receptor-1, *Olr1*)	ZFN	↓stroke susceptibility independently of BP	Liang et al. [117]
SS/JrHsdMcwi	Renin (*Ren*)	ZFN	↓BP, abnormal kidney morphology	Moreno et al. [120]
	Rag1 (recombination activating 1, *Rag1*)	ZFN	↓BP, ↓renal injury	Mattson et al. [121]
	ROMK (renal outer medullary potassium channel, *Kcnj1*)	ZFN	↓BP	Zhou et al. [122]
	Plekha7 (pleckstrin homology domain containing family A member 7, *Plekha7*)	ZFN	↓BP, ↓renal injury, ↓cardiac fibrosis	Endres et al. [123]
	HV1 (voltage-gated H^+^ channel, *Hvcn1*)	ZFN	↓BP, ↓renal injury, ↓oxidative stress	Jin et al. [124]
	CD247 (*Cd247*)	ZFN	↓BP, ↓CD3^+^ T cells, ↓renal injury	Rudemiller et al. [125]
	BNP (B-type natriuretic peptide, *Nppb*)	ZFN	↑BP, ↑cardiac hypertrophy and fibrosis, ↑renal injury	Holditch et al. [126]
	Nr2f2 (nuclear receptor subfamily 2 group F member 2, *Nr2f2*)	ZFN	↓BP, ↑left ventricular/vascular functions, ↑urinary protein	Kumarasamy et al. [127]
	Adora2b (A_2B_ adenosine receptor, *Adora2b*)	ZFN	↑BP, ↑body weight, ↓glucose clearance	Nayak et al. [128]
	Nox4 (NADPH oxidase 4, *Nox4*)	ZFN	↓BP, ↓renal injury, ↓oxidative stress	Cowley et al. [129]
	Rffl-lnc1 (a novel long-noncoding RNA)	CRISPR-Cas9	↑BP, shorter QT intervals	Cheng et al. [130]
	Resp18 (regulated endocrine-specific protein 18, *Resp18*)	ZFN	↑BP, ↑renal injury, ↓survival time	Kumarasamy et al. [131]
	Gper1 (G protein-coupled estrogen receptor 1, *Gper1*)	CRISPR-Cas9	↓BP, ↑vascular relaxation, ↓microbiotal dysbiosis	Waghulde et al. [132]
	p67^phox^ (neutrophil cytosolic factor 2, *Ncf2*)	ZFN	↓BP, ↓renal injury, ↓renal immune cell infiltration	Abais-Battad et al. [133]

## 4. Conclusions

Hypertension is a multifactorial disease; nevertheless, the majority of previous research has focused on monogenic effects under inducible hypertensive conditions such as Ang II infusion and DOCA-salt in mice. Recent advances in genome-editing techniques, however, have made it possible to create knock-out and knock-in animals more easily, efficiently, and rapidly in rats as well as in mice [140,141,142,143]. Accordingly, it is necessary to create knock-out and knock-in models with multiple mutations in different loci to mimic complex genetic backgrounds of hypertensive patients and to uncover how the genetic interactions cause hypertension. As in the case of Dahl SS [144], translation of the findings in the experimental model into human hypertension remains highly challenging. However, translational approaches to bridge the gap between humans and rodent models would be necessary for understanding genetic and molecular mechanisms of essential hypertension in the post-GWAS era. A goal of basic hypertension research using experimental models may reconstruct ‘genetically hypertensive mice/rats’ from normotensive strains, and vice versa.

## Data Availability

Not applicable.
